# The C3-C3aR axis modulates trained immunity in alveolar macrophages

**DOI:** 10.1101/2024.11.01.621042

**Published:** 2024-11-05

**Authors:** Alexander P. Earhart, Rafael Aponte Alburquerque, Marick Starick, Aasritha Nallapu, Lorena Garnica, Ayse Naz Ozanturk, Rahul Kumar Maurya, Xiaobo Wu, Jeffrey A. Haspel, Hrishikesh S. Kulkarni

**Affiliations:** 1 Division of Pulmonary and Critical Care Medicine, Washington University School of Medicine; 2 Division of Rheumatology, John T. Milliken Department of Medicine, Washington University School of Medicine; 3 Department of Medicine, University of California, Los Angeles David Geffen School of Medicine

## Abstract

Complement protein C3 is crucial for immune responses in mucosal sites such as the lung, where it aids in microbe elimination and enhances inflammation. While trained immunity – enhanced secondary responses of innate immune cells after prior exposure – is well-studied, the role of the complement system in trained immune responses remains unclear. We investigated the role of C3 in trained immunity and found that *in vivo*, trained wild-type mice showed significantly elevated pro-inflammatory cytokines and increased C3a levels upon a second stimulus, whereas C3-deficient mice exhibited a blunted cytokine response and heightened evidence of lung injury. *Ex vivo*, C3-deficient alveolar macrophages (AMs) displayed reduced chemokine and cytokine output after training, which was restored by exogenous C3 but not by C3a. Inhibiting C3aR, both pharmacologically and with a genetic C3aR knockout, prevented this restoration, indicating the necessity of C3aR engagement. Mechanistically, trained WT AMs demonstrated enhanced glycolytic activity compared to C3-deficient AMs – a defect corrected by exogenous C3 in a C3aR-dependent manner. These findings reveal that C3 modulates trained immunity in AMs through C3aR signaling, affecting cytokine production and metabolic reprogramming, and highlight a novel role for C3 in trained immunity.

## INTRODUCTION

The complement system is a crucial part of immunity, consisting of proteolytic enzymes that generate fragments which enhance antibody binding and help induce phagocytosis (Sahu et al., 2022). A central component of this system is the C3 protein, which is cleaved into its constituent components C3a and C3b upon activation (Kulkarni et al., 2018). C3b tags pathogens for phagocytosis and plays a vital role in the formation of the C3 convertase, which cleaves C5 and eventually forms the membrane attack complex that lyses targeted cells (Janssen et al., 2006). Meanwhile, C3a acts as an anaphylatoxin, binding to its cognate C3a receptor (C3aR) on and within cells, and modulates inflammatory responses (Kildsgaard et al., 2000). C3 activation has also been shown to favor an increase in glycolysis, suggesting a plausible mechanism for enhanced inflammatory activity (Friščić et al., 2021; Kang et al., 2024). Moreover, C3 is present at low levels in the bronchoalveolar lavage (BAL) fluid of uninjured mice as well as humans, indicative of localized production (Bolger et al., 2007). This local C3 production increases during injury, affecting mucosal responses to infection (Bolger et al., 2007). Additionally, the conversion of C3 to a conformationally altered C3(H_2_O) moiety increases during inflammation (Elvington et al., 2019). This C3(H_2_O) form is that which is internalized by various cell types, playing a key role in modulating survival and effector immune responses (Elvington et al., 2017; Kulkarni et al., 2019). Thus, local C3 activity plays an important role in modulating mucosal immune responses (Kulkarni et al., 2024).

A new concept of immune “memory” has recently emerged, whereby innate immune cells, particularly monocytes and macrophages, exhibit robust, enhanced inflammatory responses upon secondary stimulation after a prior insult (Quintin et al., 2012; Saeed et al., 2014). This form of “memory” is termed trained immunity and considered broadly antigen nonspecific, yet lasts several months after the initial stimulus (Netea et al., 2020). Underlying the induction of trained immunity are epigenetic and metabolic reconfigurations occurring after an initial stimulus, which prime transcriptional machinery for rapid activation after engaging secondary insults (Bekkering et al., 2014; Fanucchi et al., 2021). For instance, the bacterial and fungal cell wall component 1,3-D-β-glucan, binds the dectin-1 pattern recognition receptor, which induces metabolic changes favoring persistent glycolytic activity (Cheng et al., 2014; Earhart et al., 2023). This, in turn, promotes shifts in epigenetic modifications such as histone acetylation and methylation favoring strong pro-inflammatory gene expression upon restimulation, and can be long-lasting (Fok et al., 2019; Tercan et al., 2021). While knowledge of trained immunity is primarily systemic, there is growing evidence for site-specific effects such as in alveolar macrophages (AMs) (Chakraborty et al., 2023; Zahalka et al., 2022). Little is currently known about how the complement system affects trained immunity. Here, we sought to investigate whether the C3 protein – a key component of immune responses at mucosal sites such as the respiratory system – affects trained immunity in AMs and determine whether any effect is mediated by C3aR activity.

## RESULTS AND DISCUSSION

**Figure 1.**
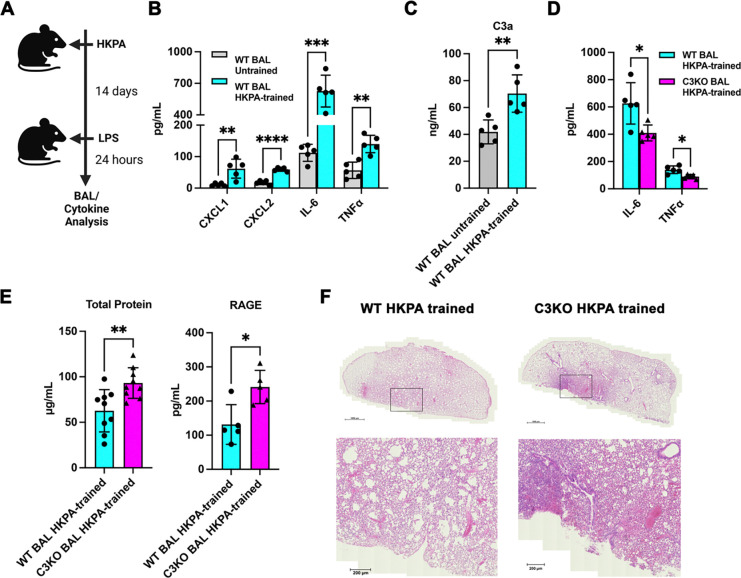
C3 deficiency predisposes to impaired pulmonary trained immunity and diminished protection from lung injury. (A) Schematic representing the training of mice via the intranasal route with heat-killed *Pseudomonas aeruginosa* (HKPA) and subsequent restimulation with lipopolysaccharide (LPS), followed by bronchoalveolar lavage (BAL) and cytokine analysis. Created with BioRender. (B) WT untrained compared against WT-trained BAL levels of CXCL1, CXCL2, IL-6, and TNFα. (C) Comparison of BAL C3a levels, similar to (B). (D) WT-trained versus C3-deficient (C3KO)-trained BAL concentrations of IL-6 and TNFα. WT-trained levels derived from (B) for comparison with C3KO-trained mice. (E) Concentrations of protein and RAGE from WT-trained versus C3KO-trained mice. (F) Representative histopathological slides of lungs showing increased tissue damage in C3KO-trained versus WT-trained mice (N=5 in each group), high power view from selected area. Data were compared with two-sided unpaired t-tests with (B, D) or without (C, E) Holm-Šidák correction for multiple hypothesis testing. Each point represents a measurement from one mouse, n = 5–10 for each group with mean ± SD shown. *p < 0.05, **p < 0.01, ***p < 0.001.

To investigate the role of C3 in pulmonary immune cell-trained immunity, we first inoculated C57BL/6J wild-type (WT) and B6.129S4-*C3*^*tm1Crr*^/J C3 knockout (C3KO) mice intranasally with heat-killed *Pseudomonas aeruginosa* (HKPA) for training or vehicle control (PBS, untrained). After 14 days, lipopolysaccharide (LPS) from *Escherichia coli* was also administered intranasally to both mouse strains to induce secondary stimulation for 24 h, followed by euthanasia and bronchoalveolar lavage (BAL) as previously described (Fig. 1A) (Sahu et al., 2023). BAL proinflammatory chemokines and cytokines (CXCL1, CXCL2, IL-6, and TNFα), total protein content (a marker of alveolar-capillary barrier disruption) and RAGE (a marker of epithelial injury), were quantified using ELISAs. Additionally, C3a, which is generated when C3 is activated and cleaved, was also measured using an ELISA specific to its neoepitope. CXCL1, CXCL2, IL-6, and TNFα were all significantly elevated in trained versus untrained WT BAL (Fig. 1B). Levels of C3a were increased in trained versus untrained WT BAL, indicating enhanced C3 activation is part of the trained immune response (Fig. 1C). The enhancement in BAL IL-6 and TNFα with training was blunted in C3-deficient mice compared to WT mice (Fig. 1D). In contrast to the blunted training response, BAL total protein and RAGE were elevated in trained C3KO versus WT mice post-LPS challenge, and alveolar damage was worse on histopathology in C3-deficient compared to WT mice (Figs. 1E, F). These results suggest C3 is important for trained immunity *in vivo*.

**Figure 2:**
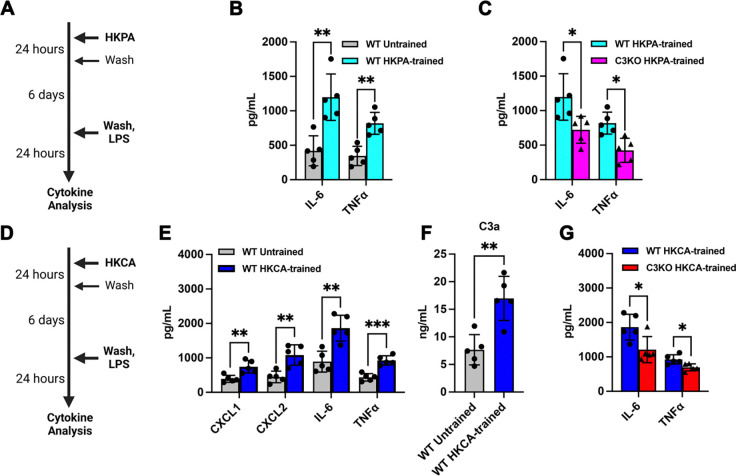
C3 deficiency results in impaired trained immune responses in ex vivo alveolar macrophages (AMs). (A) Schematic representing *in vitro* training of AMs with HKPA, with later stimulation by LPS and subsequent cytokine analysis of the supernatants. (B-C) Effects of HKPA-induced training *in vitro* on IL-6 and TNFα in supernatant from (B) WT AM, and (C) their comparison with C3KO-trained AMs. (D) Schematic representing *in vitro* training of AMs with HKCA, with subsequent restimulation by LPS and cytokine analysis of the supernatants. (E) Effects of heat-killed *Candida albicans* (HKCA)-induced training *in vitro* on CXCL1, CXCL2, IL-6 and TNFα in supernatant from WT AM. (F) Comparison of C3a levels post-HKCA training, similar to (B). (G) Comparison of IL-6 and TNFα post-HKCA training in WT versus C3KO AMs. WT-trained levels derived from (D) for comparison with C3KO-trained AMs. Data were compared with two-sided unpaired t-tests with (B,C,E,G) or without (F) Holm-Šidák correction for multiple hypothesis testing. Each point is a technical replicate made by pooling AMs from at least n=4 mice in each group, with mean ± SD shown, and each experiment was repeated twice. *p < 0.05, **p < 0.01, ***p < 0.001.

To examine how C3 affects trained immunity specifically in tissue-resident phagocytes, we set up an *ex vivo* culture system using primary alveolar macrophages (AM) from C3-deficient and wildtype mice (Gorki et al., 2022). Like the *in vivo* experiments (Fig. 1), we used HKPA to induce trained immunity in cultured AMs, followed days later by LPS challenge and analysis of cytokine secretion in the presence or absence of C3 deficiency (Fig. 2A). Supporting our *in vivo* results, HKPA was sufficient to induce trained immune responses from primary alveolar macrophages (Fig. 2B). However, C3-deficient AMs had blunted trained immune responses as measured by IL-6 and TNFα secretion (Fig. 2C). Employing heat-killed *Candida albicans* (HKCA) in place of HKPA produced similar results (Figs. 2D–G). These data suggest a specific role for C3 in AM immune training, consistent with our *in vivo* observations.

**Figure 3.**
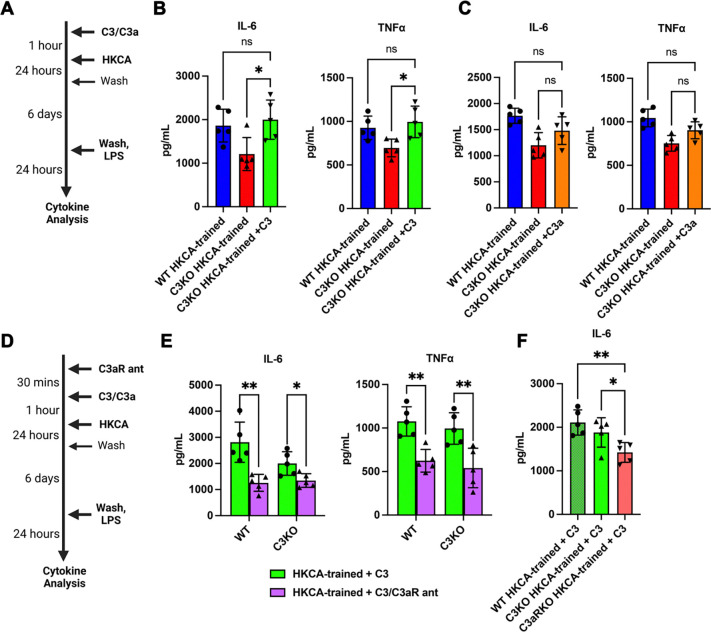
C3 uptake enhances trained immune responses in ex vivo alveolar macrophages via the C3a receptor (C3aR). (A) Schematic representing *in vitro* training of AMs with HKCA, with pre-treatment of C3 or C3a prior to induction of training, and later stimulation by LPS and subsequent cytokine analysis of the supernatants. (B) Effects of adding C3 prior to training on IL-6 and TNFα levels from C3KO AMs and their comparison with WT-trained AMs. (C) Effects of adding C3a prior to training, similar to (B). (D) Schematic representing addition of the C3aR antagonist prior to C3 treatment and *in vitro* training of AMs with HKCA, with later stimulation by LPS and subsequent cytokine analysis of the supernatants. (E) Effects of C3aR antagonism on IL-6 and TNFα levels from trained WT and C3KO AMs treated with exogenous C3. (F) Comparison of IL-6 levels post-HKCA-training in C3aR-deficient (C3aRKO), C3KO and WT AMs treated with exogenous C3. Data were compared using one way ANOVA with Dunnett’s *post hoc* tests (B,C,F) or two-sided unpaired t-testing with Holm-Šidák correction for multiple testing (D). Each point is a technical replicate made by pooling AMs from at least n=4 mice in each group, with mean ± SD shown, and each experiment was repeated twice. *p < 0.05, **p < 0.01.

To mechanistically validate the role of C3 or its products in trained immunity, we cultured C3-deficient AMs with exogenous C3 at a dose that leads to the cellular uptake of C3(H_2_O) (15 μg/mL, Fig 2A) (Elvington et al., 2017; Kulkarni et al., 2019). As a control, we incubated cells with C3a, which is not internalized (Fig. 3A) (Mogilenko et al., 2022). As measured by IL-6 and TNFα, exogenous C3 protein restored AM trained immunity to WT levels (Fig. 3B). In contrast, trained immunity was not restored by exogenous C3a, which stays outside the cell (Fig. 3C).

Upon internalization, C3 is cleaved to C3a (Elvington et al., 2017). Intracellular C3a binds to C3aR in lysosomes, and affects cytokine production in CD4^+^ T cells (Liszewski et al., 2013). To investigate whether a similar pathway could be important to trained immunity, we pre-treated AMs with a cell-permeable C3aR antagonist (SB290157) before immune training (Fig. 3D). C3aR antagonism blunted AM trained immunity in both WT and C3KO AMs that had previously been rescued with exogenous C3 (Fig. 3E). Importantly, genetic C3aR deficiency phenocopied the blunting of AM trained immune responses seen in C3-deficient cells (Fig. 3F). Altogether, these findings support a causal role for C3 in AM trained immunity via its intracellular binding of C3aR.

**Figure 4.**
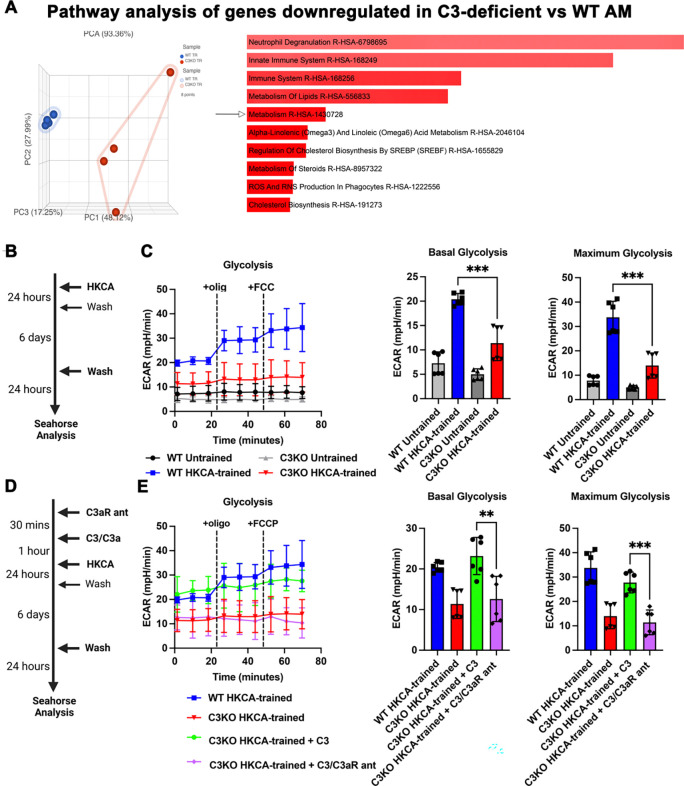
C3-C3aR axis is required for glycolysis as a part of trained immune responses in alveolar macrophages. (A) Principal component analysis (PCA, left) and EnrichR analysis of 391 genes (right, Table S1) downregulated in HKCA-trained C3KO vs WT AM by filtering genes (FDR step up ≤0.05). Arrow shows metabolism gene set in EnrichR; bars ranked by p-value. (B) Schematic representing *in vitro* training of AMs with HKCA. (C) Extracellular acidification rate (ECAR) from Seahorse analysis representing full glycolytic activity, and basal and maximum glycolysis in untrained and HKCA-trained WT and C3KO AMs. (D) Schematic representing addition of the C3aR antagonist (SB290157) prior to C3 treatment and *in vitro* training of AMs with HKCA. (E) Seahorse analysis in the presence and absence of exogenous C3 supplementation and C3aR antagonism. Each point is a technical replicate of pooled AMs from at least n=4 mice in each group, with mean ± SD shown. *p < 0.05, **p < 0.01 using an unpaired t-test.

To explore transcriptomic changes relevant to how C3 influences trained immune responses, we performed bulk RNASeq comparing trained WT to trained C3KO AMs. C3-deficiency led to differential expression of not only innate immune genes but also several genes involved in metabolism, including those relevant to glycolysis such as *gnpda1* and *aldoc* (Fig. 4A, Table S1). These metabolism-linked genes are significant because prior studies show that enhanced glycolytic metabolism is critical for trained immunity (Cheng et al., 2014). Therefore, we investigated whether the absence of C3 affects glycolytic flux in AMs (Fig. 4B). As measured by extracellular acidification rate, untrained C3-deficient AMs had comparable basal and maximum glycolytic flux compared to WT but failed to augment glycolysis upon training with HKCA (Fig. 4C). We next tested the ability of exogenous C3 to rescue the induction of training-associated glycolysis in C3-deficient AMs (Fig. 4D). Exogenous C3 returned glycolysis induction to the level of trained WT cells, suggesting intracellular processing of C3 is important for this effect (Fig. 4E). Like cytokines (Fig. 3), inhibiting C3aR prevented exogenous C3 from rescuing glycolysis induction during immune training (Fig. 4E).

Collectively, these data indicate that C3 makes a mechanistic contribution to trained immunity in AMs and implicate engagement of the C3aR as part of the pathway. Strengths of our study include *in vitro* and *in vivo* approaches to inducing trained immunity, orthogonal endpoints to quantify immune training (cytokine and metabolic), and mechanistic gain- and loss-of-function experimental approaches. Future work involves identifying the downstream effectors of C3 and C3aR in the AM training process and their connection to immunometabolism. The results may have clinical implications since trained immunity is linked to vaccine effectiveness (for example, with respect to the Bacillus Calmette-Guérin vaccine) and overall immune resilience (Arts et al., 2018; Kleinnijenhuis et al., 2015). Moreover, humans with C3 deficiency demonstrate impaired responses to vaccination (Kim et al., 2018; Pekkarinen et al., 2015). It is tempting to speculate that local augmentation of C3 and/or C3aR activity – could enhance vaccine effectiveness for hard-to-vaccinate pathogens like *Mycobacterium tuberculosis*.

## MATERIALS AND METHODS

Details including mouse strains, design, models of trained immunity, readouts, and statistical analysis are described as per the ARRIVE guidelines and included in Supplementary Material.

## Supplementary Material

Supplement 1

Supplement 2

## Data Availability

Sequencing data have been deposited in GEO under the accession code GSE281001 at https://www.ncbi.nlm.nih.gov/geo/query/acc.cgi?acc=GSE281001.
