# Supplementary material for: The C3-C3aR axis modulates trained immunity in alveolar macrophages
Source: bioRxiv. 2024 Nov 5:2024.11.01.621042. Preprint. [Version 2] doi: 10.1101/2024.11.01.621042 (PMC11565986; doi:10.1101/2024.11.01.621042)
Supplement: Supplement 1 [file media-1.pdf]

SUPPLEMENTARY MATERIAL

TABLES

Table S1. Table of pathways based on Reactome analysis with a list of overlapping genes, when comparing downregulated transcripts between trained C3-deficient and wildtype primary alveolar macrophages.

METHODS

Experimental model details

Mice

All animal studies were conducted on an approved IACUC protocol. C57BL/6J (RRID:IMSR_JAX:000664, termed wild-type, or WT) and B6.129S4-$C3^{tm1Crr}$/J (RRID:IMSR_JAX:003641, termed C3-deficient, or C3KO) mice were obtained from Jackson Laboratory (Bar Harbor, ME, USA). The mice were maintained as groups in our animal housing facility at the Washington University in St. Louis School of Medicine with a 12 h light-dark cycle, with temperatures between 22-23°C and humidity of 68-72%. C3aR-deficient (termed C3aRKO) mice were provided by Dr. Rick Wetsel at the University of Texas in Houston, were bred in house and have been reported previously by us (Kildsgaard et al., 2000; Sahu et al., 2023).

Experimental Design

The ARRIVE guidelines were followed for reporting of in vivo experiments, and are reported throughout the Methods and Figure Legends. Female and male mice between the ages of 8 and 16 weeks of age were used in these experiments and were age-matched in each experiment. Sample size was determined based on prior studies on acute lung injury, a minimum of 5 mice were used from each genotype (Sahu et al., 2023). Pre-established exclusion criteria for mice included being pregnant or injured (i.e., unanticipated pre-existing

wounds from co-housing). Each experiment was repeated at least twice, and both biological and technical replicates were included. No sample or data point from the analysis was omitted.

In vivo trained immunity in mice

WT and C3KO mice were administered heat-killed *Pseudomonas aeruginosa* (HKPA, Pa57-15, $1x10^5$ CFU/mouse) (Sahu et al., 2023) or PBS vehicle control intranasally. After 14 days, LPS (*E. coli* O111:B4, Millipore Sigma, cat #: L4391, 10 μg/mouse) or PBS was also intranasally given to these mice. 24 h later, mice were anesthetized with 1.25% tribromoethanol (approx. 125-250 mg/kg) via intraperitoneal injection followed by cervical dislocation for euthanasia. Bronchoalveolar lavage (BAL) was performed by inserting a 20G x 1” Surflo IV catheter into the trachea and instilling approx. 0.5 mL PBS + protease inhibitor (Halt™ Protease and Phosphatase Inhibitor Single-Use Cocktail (100X), Thermo, cat #: 78444) into the lungs. The fluid was then collected, followed by centrifugation at 3,000 rpm for 3 min at 4°C for separating the cell pellet and supernatant, which was then stored at -20°C until later use.

Lung histology

Mouse lungs were inflated with 10% formalin prior to removal and placed in 10% formalin for up to 72 h. Lungs were then washed in water and dehydrated by submerging and shaking on a plate at 200 rpm for 1 hour each in a series of steps with 30%, 50%, and finally 70% ethanol then stored at 4°C until paraffin embedding, slicing, placement on slides, and H&E staining.

Alveolar macrophage (AM) isolation and maintenance

Ex vivo AM were cultured as per a previously published protocol (Gorki et al., 2022; Zahalka et al., 2022). To obtain AM, BAL was performed at least 4 times per mouse, followed by centrifugation at 3,000 rpm for 3 min at 4°C. The resulting cell pellet was resuspended in AM

media (RPMI +10% FBS [Biowest, cat #: S1620], 1% penicillin+streptomycin, 1 µM Rosiglitazone [Millipore Sigma, cat #: R2408], 10 ng/mL mouse TGF-β1 [BioLegend, cat #: 781804], and 30 ng/mL mouse GM-CSF [PeproTech, cat #: 315-03-100UG]), plated on 25 mm round dishes, and incubated at 37°C + 5% CO_2 for 24 h. Media was then removed, and cells were washed twice with warm PBS to removed unwanted cells and debris. AM media was added again, and cells allowed to grow for at least 7 days. After this, cells were washed again with warm PBS then incubated for 15-20 min with Accutase (ThermoFisher, cat #: MT25058CI) to gently remove them from the plates. They were then centrifuged at 500g for 5 min at 4°C, resuspended in AM media, then placed in T25 flasks and incubated at 37°C + 5% CO_2. Resulting AMs were passaged every 7 days for a maximum of 25 passages (Gorki et al., 2022).

Induction of trained immune responses in AM in vitro.

AMs were plated at $1x10^5$ cells/well in 96-well flat bottom tissue culture-treated plates and incubated at 37°C +5% CO_2 for 30 min to 1 h to promote adherence. Each well was then washed with PBS, followed by the addition of "trained" AM media supplemented with HKPA or heat-killed *Candida albicans* (HKCA, $1x10^4$ CFU HKPA or HKCA/well, Invitrogen, cat #: tlrl-hkca) or a matched volume of PBS as a vehicle control for "untrained" wells. Plates were incubated for 24 h at 37°C + 5% CO_2, washed with PBS, then rested for 6 days in AM media, followed by secondary stimulation with LPS (10 ng/mL, *E. coli* O111:B4, Millipore Sigma, cat #: L4391). After 24 h, supernatants were collected and frozen at -20°C until later use.

Exogenous treatment of AM with C3 and C3a

In order to investigate the effects of C3 uptake or C3a individually on training, either C3 (15 µg/mL, CompTech, cat #: M113) or C3a (10 µg/mL, CompTech, cat #: A118) were added to wells 1 h prior to training of WT, C3KO, or C3aRKO AMs with HKCA. To examine whether C3/C3a affects training via the C3a receptor (C3aR), wells were supplemented with 200 nM of

C3aR antagonist SB290157 (Millipore Sigma,cat #: SML1192) 30 min prior to either training with HKCA, or addition of exogenous C3 or C3a followed by training 1 h later.

Quantification of chemokines and cytokines

Untrained and trained BAL and AM supernatants obtained as described above were thawed to room temperature from -20°C. Concentrations of CXCL1, CXCL2, IL-6, and TNFα, or also RAGE and total protein in BAL, were determined via competitive ELISA plates (R&D Systems Inc., Minneapolis, MN, USA) or Milliplex plates (Millipore Sigma, St. Louis, MO, USA) according to manufacturer's instructions at 1:2 or 1:4 dilution. Sandwich ELISAs were then read on an EPOCH microplate reader via optical density measurement at 450 nm wavelength. Multiplex ELISA plates (Millipore Sigma, St. Louis, MO, USA) were read using a Bio-Rad Luminex 100 multiplex system.

Quantification of C3a-neo

The protocol for measuring C3a-neo in the supernatant was adapted from a previously published protocol measuring it in the serum (Pagano et al., 2009). ELISA plates (96-well flat-bottom; #3855, Thermo Fisher Scientific) were coated with Mouse C3a Capture Antibody (1:250; 100μl per well of PBS; Purified Rat Anti-Mouse C3a, Cat# 558250, BD Pharmingen) overnight at 4°C. After washing three times with a solution of 0.05% Tween 20 in PBS, the plate was blocked with 1% bovine serum albumin (BSA; #A7906, Sigma-Aldrich) at room temperature (RT) for 1 h. Plates were washed again and samples diluted at 1:4 in 1% BSA/PBS solution were added (100 μl per well). Standard curve, made from purified mouse C3a (Purified Mouse C3a Protein, Cat# 558618 BD Pharmingen) was used from 50 to 3.125 ng/ml. Samples and purified protein were incubated at RT for 2 h. The plates were subsequently washed three times and Mouse C3a Detection Antibody (1:1000; 100μl per well in 1% BSA/PBS; Biotin Rat Anti-Mouse C3a, #558251, BD Pharmingen) was diluted in 1% BSA. After another three washes,

samples were incubated with Streptavidin HRP-conjugated (100µl per well; 1:200 dilution, #DY998, R&D Systems) for 30 min at RT. After three washes, TMB Color Substrate (#DY999, R&D Systems) was added at 100 µl per well and incubated at RT for 5 min. The reaction was stopped by addition of 1 M sulfuric acid (50 µl per well; #DY994, R&D Systems), and optical density was measured at 450 nm (Epoch Microplate Spectrophotometer, BioTek).

Cell metabolism

To examine a possible mechanistic basis for differences in trained immune responses in WT, C3KO, and C3aRKO AMs, cells were plated at $1x10^5$/well of an XFe24-well cell culture plate (Agilent Technologies, Santa Clara, CA, USA) and trained in AM media with or without C3/C3a, or SB290157 and subsequently washed and rested as described above. After 6 days, cells were restimulated with LPS (10 ng/mL) for 24 h, washed, then left in mitochondrial stress test buffer (Agilent XF DMEM media supplemented with 10 mM glucose, 2 mM glutamine, and 1 mM sodium pyruvate, pH 7.4) at 37°C with no CO_2 for 1 h. Glycolysis by means of extracellular acidification rate (ECAR) via lactate production in the supernatant was analyzed using an Agilent Seahorse XFe24 analyzer. Three time points were measured each of stable basal glycolysis; after addition of 2.5 µM oligomycin and following the injection of 2 µM FCCP for maximal glycolysis; and was performed according to manufacturer instructions.

RNA sequencing

After training C3-deficient and WT alveolar macrophages as described, RNA was extracted using an RNeasy Plus kit (Qiagen, Cat #: 74134). Samples were sent for RNA-Sequencing with polyA selection. Samples were prepared according to library kit manufacturer's protocol, indexed, pooled, and sequenced on an Illumina NovaSeq 6000. Basecalls and demultiplexing were performed with Illumina's bcl2fastq software and a custom python demultiplexing program with a maximum of one mismatch in the indexing read. RNA-seq reads

were then aligned to the Ensembl release 101 primary assembly with STAR version 2.7.9a (Dobin et al., 2013). Gene counts were derived from the number of uniquely aligned unambiguous reads by Subread:featureCount version 2.0.3 (Liao et al., 2014). Isoform expression of known Ensembl transcripts were quantified with Salmon version 1.5.2 (Patro et al., 2017). Sequencing performance was assessed for the total number of aligned reads, total number of uniquely aligned reads, and features detected. The ribosomal fraction, known junction saturation, and read distribution over known gene models were quantified with RSeQC version 4.0 (Wang et al., 2012).

All gene counts were then imported into the R/Bioconductor package EdgeR (Robinson et al., 2010) and TMM normalization size factors were calculated to adjust for samples for differences in library size. Ribosomal genes and genes not expressed in the smallest group size minus one samples greater than one count-per-million were excluded from further analysis. The TMM size factors and the matrix of counts were then imported into the R/Bioconductor package Limma (Ritchie et al., 2015). Weighted likelihoods based on the observed mean-variance relationship of every gene and sample were then calculated for all samples with the voomWithQualityWeights (Liu et al., 2015) function and were fitted using a Limma generalized linear model with additional unknown latent effects as determined by surrogate variable analysis (SVA) (Leek and Storey, 2007). The performance of all genes was assessed with plots of the residual standard deviation of every gene to their average log-count with a robustly fitted trend line of the residuals. Differential expression analysis was then performed to analyze for differences between conditions and the results were filtered for only those genes with Benjamini-Hochberg false-discovery rate adjusted p-values less than or equal to 0.05.

For each contrast extracted with Limma, global perturbations in known Gene Ontology (GO) terms, MSigDb, and KEGG pathways were detected using the R/Bioconductor package

GAGE (Luo et al., 2009) to test for changes in expression of the reported log 2 fold-changes reported by Limma in each term versus the background log 2 fold-changes of all genes found outside the respective term. The R/Bioconductor package heatmap3 (Zhao et al., 2014) was used to display heatmaps across groups of samples for each GO or MSigDb term with a Benjamini-Hochberg false-discovery rate adjusted p-value less than or equal to 0.05. Perturbed KEGG pathways where the observed log 2 fold-changes of genes within the term were significantly perturbed in a single-direction versus background or in any direction compared to other genes within a given term with p-values less than or equal to 0.05 were rendered as annotated KEGG graphs with the R/Bioconductor package Pathview (Luo and Brouwer, 2013).

To find the most critical genes, the Limma voomWithQualityWeights transformed log 2 counts-per-million expression data was then analyzed via weighted gene correlation network analysis with the R/Bioconductor package WGCNA (Langfelder and Horvath, 2008). Briefly, all genes were correlated across each other by Pearson correlations and clustered by expression similarity into unsigned modules using a power threshold empirically determined from the data. An eigengene was then created for each de novo cluster and its expression profile was then correlated across all coefficients of the model matrix. Because these clusters of genes were created by expression profile rather than known functional similarity, the clustered modules were given the names of random colors where grey is the only module that has any pre-existing definition of containing genes that do not cluster well with others. These de-novo clustered genes were then tested for functional enrichment of known GO terms with hypergeometric tests available in the R/Bioconductor package clusterProfiler (Yu et al., 2012). Significant terms with Benjamini-Hochberg adjusted p-values less than 0.05 were then collapsed by similarity into clusterProfiler category network plots to display the most significant terms for each module of hub genes in order to interpolate the function of each significant module. The information for all clustered genes for each module were then combined with their respective statistical

significance results from Limma to determine whether or not those features were also found to be significantly differentially expressed. The data was subsequently processed using Partek Flow and pathway analysis was done using EnrichR (Chen et al., 2013; Kuleshov et al., 2016; Xie et al., 2021).

Statistical analyses

Direct comparisons of two isolated groups were analyzed via two-sided unpaired t-test, while multiple two group comparisons were done via t-test with the Holm-Šidák correction to control for the family-wise error rate. Analyses of three or more groups against each other were performed using the one-way analysis of variance with Dunnett's *post hoc* tests to correct for multiple comparisons. *P* values less than 0.05 were considered statistically significant. Statistical analyses were performed with GraphPad Prism 10.0, and independently with R. Data are shown as individual measurements with mean ± SD, while no outliers have been removed.

Data

The RNASeq data that support the findings have already been deposited in NCBI GEO and will be made publicly available at the time of publication. The data are available from the corresponding author prior to publication upon reasonable request.
